# Combining Traditional and Molecular Techniques Supports the Discovery of a Novel *Legionella* Species During Environmental Surveillance in a Healthcare Facility

**DOI:** 10.3389/fmicb.2022.900936

**Published:** 2022-06-13

**Authors:** Luna Girolamini, Maria Rosaria Pascale, Marta Mazzotta, Simona Spiteri, Federica Marino, Silvano Salaris, Antonella Grottola, Massimiliano Orsini, Sandra Cristino

**Affiliations:** ^1^Department of Biological, Geological, and Environmental Sciences, University of Bologna, Bologna, Italy; ^2^European Society of Clinical Microbiology and Infectious Diseases (ESCMID) Study Group for Legionella Infections (ESGLI), Basel, Switzerland; ^3^Department of Civil, Chemical, Environmental, and Materials Engineering, University of Bologna, Bologna, Italy; ^4^Department of Specialty, Diagnostic and Experimental Medicine, University of Bologna, Bologna, Italy; ^5^Regional Reference Laboratory for Clinical Diagnosis of Legionellosis, Molecular Microbiology and Virology Unit, University Hospital-Policlinico Modena, Modena, Italy; ^6^Laboratory of Microbial Ecology and Genomics of Microorganisms, Istituto Zooprofilattico Sperimentale delle Venezie, Legnaro, Italy

**Keywords:** *mip* sequencing, *rpoB* sequencing, MALDI-TOF MS, water distribution system, whole-genome sequencing (WGS)

## Abstract

*Legionella* surveillance plays a significant role not only to prevent the risk of infection but also to study the ecology of isolates, their characteristics, and how their prevalence changes in the environment. The difficulty in *Legionella* isolation, identification, and typing results in a low notification rate; therefore, human infection is still underestimated. In addition, during *Legionella* surveillance, the special attention given to *Legionella pneumophila* leads to an underestimation of the prevalence and risk of infection for other species. This study describes the workflow performed during environmental *Legionella* surveillance that resulted in the isolation of two strains, named 8cVS16 and 9fVS26, associated with the genus *Legionella*. Traditional and novel approaches such as standard culture technique, MALDI-TOF MS, gene sequencing, and whole-genome sequencing (WGS) analysis were combined to demonstrate that isolates belong to a novel species. The strain characteristics, the differences between macrophage infectivity potential (*mip*), RNA polymerase β subunit (*rpoB*), and reference gene sequences, the average nucleotide identity (ANI) of 90.4%, and the DNA–DNA digital hybridization (dDDH) analysis of 43% demonstrate that these isolates belong to a new *Legionella* species. The finding suggests that, during the culture technique, special attention should be paid to the characteristics of the isolates that are less associated with the *Legionella* genus in order to investigate the differences found using more sensitive methods. The characterization of the two newly discovered isolates based on morphological, biochemical, and microscopic characteristics is currently underway and will be described in another future study.

## Introduction

*Legionella* spp. are gram-negative intracellular pathogenic bacteria, are ubiquitous in water and soil, and are represented by more than 66 species, some of which are potentially capable to cause a severe form of pneumonia, called Legionnaires' disease (Jomehzadeh et al., 2019; Parte et al., 2020). *Legionella pneumophila* (*Lp*) is the most common infectious agent involved in Legionnaires' disease and consists of 15 different serogroups. *Lp* serogroup 1 (*Lp*1), according to epidemiological data, is mostly associated with human infections (European Centre for Disease Prevention Control, 2021; Rota et al., 2021). Nevertheless, other *Legionella* non-*pneumophila* species (non-*Lp* species) (i.e., *L. anisa, L. rubrilucens*, or *L. longbeachae*) are responsible for human infections that are typically acquired through inhalation of contamination aerosol (Muder and Yu, 2002; Matsui et al., 2010; Cunha et al., 2016; European Centre for Disease Prevention Control, 2021; Rota et al., 2021).

Artificial water distribution systems (WDSs) are considered the sites most associated with *Legionella* spp. proliferation and spread (Mercante and Winchell, 2015). The presence of biofilm, water stagnation due to low water flow, dead branches, or old pipelines are factors that make the WDS's facilities (e.g., large public or private buildings, companies, hospitals, or health facilities) a potential risk to the dissemination of *Legionella* spp. (Di Pippo et al., 2018; Nisar et al., 2020a,b; Totaro et al., 2020).

The main attention is given to the WDS of hospitals and healthcare facilities (HCF), where *Legionella* spp. contamination is considered a high-risk factor due to the presence of elderly or immunocompromised patients who are more susceptible to the infection (Spagnolo et al., 2013). Legionnaires' disease in Italy, in 2020, amounted to 34.3 cases per million inhabitants; therefore, *Legionella* colonization of hospitals or communities WDS represents one of the main public health concerns (Kyritsi et al., 2018; Rota et al., 2021; Brunello et al., 2022).

Starting from the epidemiological data, different countries have developed specific guidelines and promoted environmental monitoring programs in order to prevent and control *Legionella* infections, following the Water Safety Plan (WSP) approach, introduced by the World Health Organization (WHO) (World Health Organization, 2007). Through this approach, an ongoing *Legionella* surveillance program is suggested, also in the absence of cases, to undertake the most appropriate control and prevention measures.

The recent European Union (EU) Directive 2020/2184 suggests the prevention approach. This directive will be transposed in all the EU countries by January 2023 (European Parliament the Council of the European Union, 2020). In this directive, *Legionella* has been introduced as a new microbiological parameter for the evaluation of the drinking-water quality, highlighting the importance of the environmental monitoring of the WDS, starting from the water supply until the consumers' outlets.

Moreover, to achieve control of *Legionella* proliferation, the national and international directives point out several disinfection strategies, based on chemical disinfectants (i.e., chlorine dioxide, monochloramines, and hydrogen peroxide) and physical treatments [i.e., ultraviolet (UV) light and hot temperature treatments]. All of them have shown some advantages and disadvantages (Mcdonnell and Russell, 1999; Richardson et al., 2007; Lin et al., 2011; Mancini et al., 2015; Girolamini et al., 2019). However, some authors have shown how these treatments, especially the use of chemical disinfectants, could select resistant strains and introduce some changes in the bacterial genome. Therefore, the use of diagnostic methods able to follow the ecological and adaptive isolates changes should be the main objective to arise also during *Legionella* environmental surveillance (Jakubek et al., 2013; Girolamini et al., 2021b). Certainly, this aspect is strictly correlated with the sensitivity and specificity of detection and identification methods already available and in use. In recent times, the most common methods for *Legionella* isolation and identification in routinely environmental surveillance remain the culture technique and the latex agglutination test, despite the molecular approach as sequence-based typing (SBT) and subgrouping scheme based on monoclonal antibodies (MABs) for *Lp* (Helbig et al., 1997, 2002; Gaia et al., 2005; Ratzow et al., 2007) and macrophage infectivity potentiator (*mip*) for non-*Lp* species (Ratcliff et al., 1998; Ko et al., 2002; Pascale et al., 2021) are consolidated. Despite several approaches that have been developed through time, most of them are applied only when clusters or outbreaks occur and are carried out only by specialized or national reference laboratories.

The limits of all the methods, previously cited, are summarized as follows: (i) long incubation time of culture technique (at least 10 days); (ii) the agglutination test that is not able to recognize all *Legionella* species, showing also mis-identification or false-negative results; (iii) MABs, that can type only *Lp* and in particular for subtyping of *Lp*1, available to the national reference laboratories; (iv) SBT technique and gene sequencing that permits compare only some gene or short fragments of gene size (Helbig et al., 2002; Gaia et al., 2005; Orsini et al., 2011; Lück et al., 2013; Walker and McDermott, 2021).

Consequently, only in recent years with the data returned by whole-genome sequencing (WGS), it has been possible to obtain the most complete genome information, improving isolate typing as well as functional and drug susceptibility response. Moreover, the WGS analysis has opened new scenarios for the reconstruction of infection spread, establishing a correct relationship between environmental and clinical strains during the epidemiological investigation (Quainoo et al., 2017). However, even with its great usefulness, it is not routinely used to support environmental surveillance. Despite the high operational costs, the WGS pipelines could potentially reduce overall costs for the hospitals as well as in all facilities' practices through savings of indirect costs (Quainoo et al., 2017).

In the context of *Legionella* environmental monitoring, the primary goal is to improve WSPs, quantify the risk level, and identify isolates. Moreover, also during routine environmental surveillance, it is possible to isolate and characterize novel new *Legionella* species, suggesting how the water microflora is subject to continuous changes during the time and in response to the treatment (physical, chemical, and functional) undertaken (Li et al., 2017).

In this study, we described the workflow applied during *Legionella* environmental surveillance conducted for 10 years in an HCF that led to the isolation of a novel *Legionella* species. The knowledge acquired during the surveillance period regarding the WDS, the disinfection treatment, and the level of contamination were associated with traditional techniques (i.e., culture and latex agglutination test) as well as the innovative matrix-assisted laser desorption ionization–time-of-flight mass spectrometry (MALDI-TOF MS) technique, gene sequencing, and WGS analysis. All results obtained confirm the presence in HCF of two isolates not previously detected.

## Materials and Methods

### Characteristics of Healthcare Facility's Water Distribution System

The HCF involved in this study is a long-term care facility, built-in 2011, and made up of 78 dislocated inpatient rooms on three floors for a total of 120 beds.

The *Legionella* environmental surveillance program was started in 2012 with an elaboration of a *Legionella* risk assessment plan according to the Italian and Regional Guidelines (Italian National Institute of Health, 2015; Emilia-Romagna Region, 2017). Briefly, the *Legionella* monitoring was carried out two times a year, usually in the spring/summer and fall/winter. The *Legionella* concentration above the risk level as suggested by guidelines requires further sampling of positive samples (Italian National Institute of Health, 2015; Emilia-Romagna Region, 2017).

**Figure 1:**
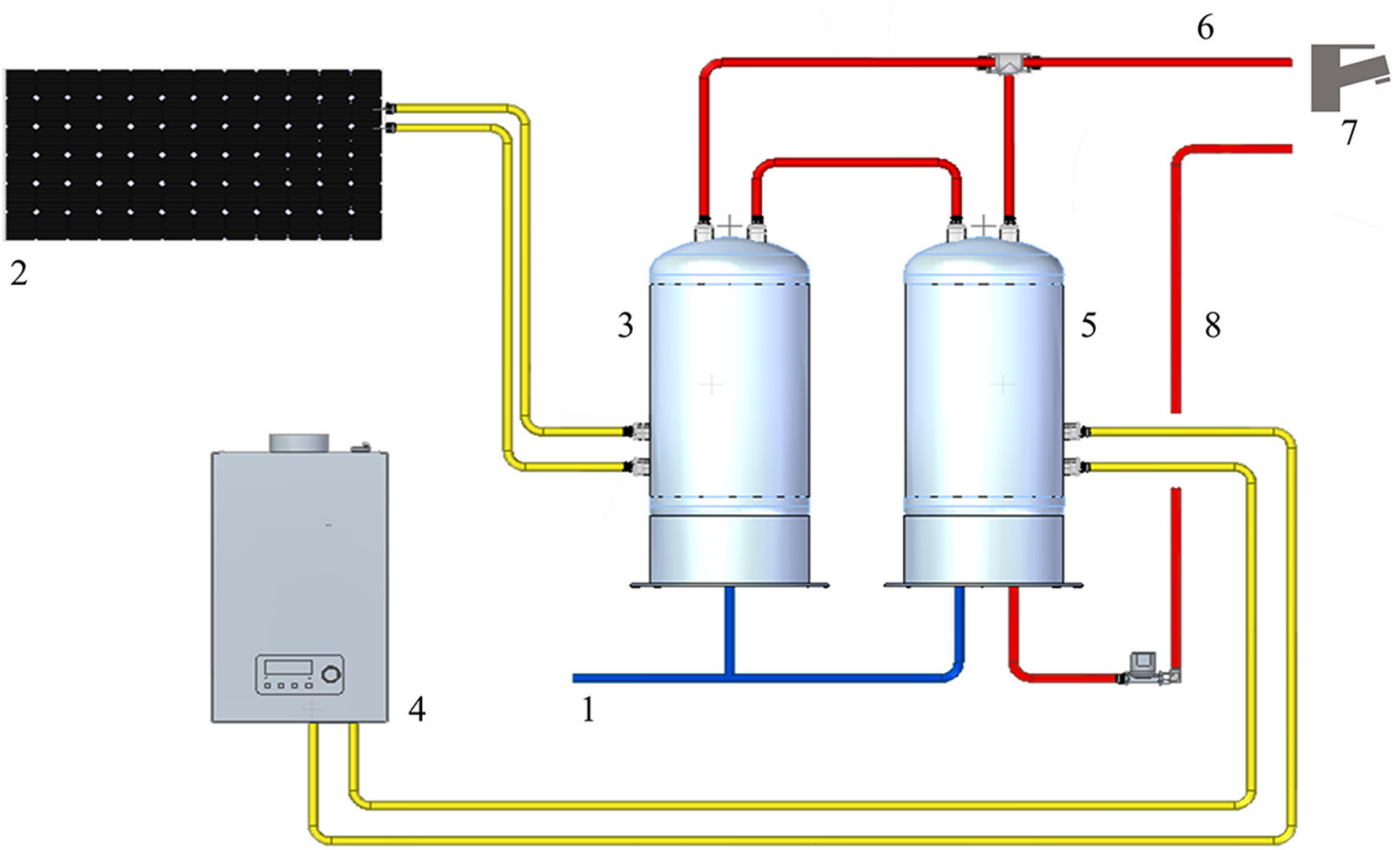
Representation of the HCF water distribution system: 1: cold-water supply; 2: solar panel; 3: solar heater water tank; 4: gas water heater; 5: heater water tank; 6: hot water output; 7: water distal outlets; 8: hot water return line.

The WDS consists of cold water derived from municipal water, heated by two water heaters in parallel, one of them connected to the solar panel. The temperature of hot water at the supply outlets was about 50°C. The hot water system is treated with hydrogen peroxide and silver salt-based disinfectant (H_2_O_2_/Ag^+^). The continuous disinfection treatment provides a residual concentration to the distal outlets around 20 mg/L. The WDS schematic (Figure 1) was developed with Solid Edge 2022 V222.00.02.03 (Siemens Digital Industry Software Inc.).

### Sample Collection

According to the *Legionella* risk assessment plan, hot- and cold-water samples were collected every 6 months. The sampling points were chosen in accordance with Italian Guidelines (Italian National Institute of Health, 2015), considering the building size, the number of inpatient rooms, the risk level of patients, and workers' exposure to bacteria, other than the facility's epidemiological data. All of these data are reported in the *Legionella* WSP developed since 2012.

Staring from the technical room, and following the hot and cold WDS, for each floor, four samples were chosen as follows: three hot water samples in the vicinity of, mid-way to, and away from the technical room, and one cold-water sample away from the water supply point.

A total of 30 sampling points were identified between the technical room (water supply point, hot water output, hot water return line, and hot water storage tanks) and HCF inpatient rooms (including showers, sinks, and toilet showerheads) and staff facilities. The criterion of rotation between inpatient rooms was applied.

Two liters of hot or cold water for each sample was collected in post-flushing modality (2 min), following the Italian National Unification and European Committee (UNI EN) International Standard Organization (ISO) 19458:2006 (EN ISO 19458:2006 Water quality - Sampling for microbiological analysis, 2006). During sampling, temperature and disinfectant residue values were measured and reported in distal outlets. The analysis was carried out on the same day of sampling.

### Microbiological Analysis and Isolates Characterization

The *Legionella* isolation was performed using a standard culture technique in accordance with ISO 11731:2017 (International Organization for Standardization, 2017). Briefly, for the enumeration of *Legionella*, different aliquots of the sample (from 200 to 100 μl), which comes from filtered water (untreated), heat- and acid-treated, were seeded on glycine–polymyxin B–vancomycin–cycloheximide (GVPC) selective agar (Thermo Fisher Scientific, Diagnostic, Ltd., Basingstoke, UK) and incubated at 35 ± 2°C with 2.5% of CO_2_. The culture required a minimum of 10 for up to 15 days. Every 2 days, the plates were examined and the presumptive colonies were enumerated and sub-cultured on buffered charcoal yeast extract (BCYE) agar with and without L-cysteine (Cys+) and L-cysteine (Cys–) (Thermo Fisher Scientific, Diagnostic, Ltd., Basingstoke, UK). The *Legionella* colonies, growth only on BCYE Cys+, were identified using the *Legionella* latex agglutination test kit differentiating between *Lp*1, and *Lp* serogroups 2–14 (*Lp*2-14) and seven species of non-*Lp* species (Thermo Fisher Scientific, Ltd. Basingstoke, UK), based on manufacturing instructions.

### MALDI-TOF MS Analysis

The isolates grown on BCYE Cys+, which returned positive or negative results for the *Legionella* agglutination test, were also analyzed by the MALDI Biotyper system (Bruker Daltonik GmbH, Bremen, Germany) as previously described (Pascale et al., 2020). Briefly, a fresh colony (24–48 h of incubation) was directly spotted in duplicate onto a MALDI Biotyper target plate, overlaid with 1 μl of the MALDI Biotyper matrix solution, and left to air dry before the next step. Spectra acquisition and processing were performed using the Microflex LT mass spectrometer (2,000–20,000 Da, linear positive mode) and the MALDI Biotyper Compass 4.1 software, whose library (version BDAL 7854) included the spectra of 39 *Legionella* strains.

The data were interpreted in accordance with the manufacturer's instructions. Briefly, when the instrument returned a log score ≥2.0 (“high confidence level”), the isolates were identified at the species level, while the genus was assigned for scores between 1.7 and 1.99 (“low confidence level”). In the presence of a score between 0.00 and 1.69, the isolates were considered as “not identified.”

A dendrogram based on hierarchical clustering analysis (HCA) of MALDI Biotyper spectra was developed using the MALDI Biotyper Compass Explorer software to generate a tree-like structure able to link the *Legionella* strains to each other using a linkage algorithm.

### Sequencing of *mip* and *rpoB* Genes for *Legionella* Identification

InstaGene Matrix (Bio-Rad, Hercules, CA, USA) was used for DNA extraction that was quantified by Qubit fluorometer (Thermo Fisher Scientific, Paisley, UK).

*mip* and *rpoB* genes were used to perform the identification of the isolates according to Ratcliff et al. (1998), Ko et al. (2002), and Pascale et al. (2021). PCR products were visualized by electrophoresis on 2% agarose gel. BigDye kit was used for the sequencing reaction, and the sequences were analyzed on ABI PRISM 3100 Genetic Analyzer (Applied Biosystems, Foster City, CA, USA). Raw sequencing data were assembled using CLC Main Workbench 7.6.4 software (QIAGEN, Redwood City, CA, USA). Furthermore, the *mip* and *rpoB* gene sequence comparison was performed using the Basic Local Alignment Search Tool (BLAST) search on the database of the National Center for Biotechnology Information (NCBI) and the database developed by the European Working Group for *Legionella* Infections [(EWGLI), renamed in ESCMID Study Group for *Legionella* Infections (ESGLI) from 2011]. The identification at the species level, considering the *mip* gene, was performed on the basis of an identity score between 98 and 100% compared to the sequences in the database (Fry et al., 2007) and considering the intervals variation of interspecies and intraspecies previously described by Ratcliff et al. (1998).

As reported in Ko et al. (2002), Adékambi et al. (2009), and Pascale et al. (2021) regarding the *rpoB* gene sequence, the threshold used for the identification was fixed at a 95% similarity percentage.

### Phylogenetic and Allelic Diversity Analysis

To estimate the relationship between the isolates found and the strictly related *Legionella* species, a multiple sequence alignment (MSA) and a concatenated phylogenetic tree were developed on the *mip* and *rpoB* gene sequences. Manual editing was performed on the sequences, if required, trimming them to the same length as the reference sequence. The tree was built using software implemented in Geneious Prime's genome browser (Geneious Prime 2022.0.2; http://www.geneious.com) maintaining the default settings (Kearse et al., 2012). Through the MUSCLE algorithm, the nucleotide sequences were aligned (Edgar, 2004). FastTree (Price et al., 2010), a tool to deduce the approximate maximum likelihood of phylogenetic trees, was used to pass the resulting MSA. Jukes–Cantor was used, by FastTree, as a genetic distance model, and the Shimodaira–Hasegawa test was utilized to estimate the reliability of each split in the tree (default parameters) (Guindon et al., 2010). As in a cladogram, the lengths of the branches have been converted to be equal. Branch labels display the substitutions by the site.

### Identification of *Legionella* by Whole-Genome Sequencing (WGS)

One hundred nanograms of DNA was used to prepare the library for the next-generation sequencing (NGS) carried out by Illumina Nextera XT DNA Library Preparation kit (Illumina, New England Biolabs, Ipswich, MA, USA). The Illumina NextSeq 500 platform (2 × 250 paired-end reads) was used to perform the sequencing.

Raw reads were assembled using TORMES v.1.2.0 (Quijada et al., 2019), an automated pipeline for whole bacterial genome analysis, using the default parameters. TORMES performed a sequence quality filtering (PRINSEQ v. 0.20.4) and a *de novo* genome assembly (SPAdes v. 13.4.1) (Bankevich et al., 2012). The generated contigs were passed to CSAR v1.1.1 (Chen et al., 2018), a scaffolding tool able to order and orient the contigs of the given draft genome based on one or more reference genomes of a related organism. *Legionella* sp. PC1000 (NZ_CP059400.1) was selected as a reference sequence, based on the output of KmerFinder (Hasman et al., 2014; Larsen et al., 2014; Clausen et al., 2018) that predicts prokaryotic species based on the number of overlapping *k*-mers, i.e., 16-mers, between the query genome and genomes in a reference database (NCBI). A further refinement was carried out by remapping the reads on the CSAR scaffolds, using the Geneious Prime 2022.0.2 software (http://www.geneious.com) (Kearse et al., 2012). Benchmarking Universal Single-Copy Orthologs (BUSCO) v.5.0.0 (Seppey and Manni, 2019) was performed to evaluate the completeness of the two genome assemblies. The final draft genomes were submitted to the GenBank requiring the annotation by the NCBI Prokaryotic Genome Annotation Pipeline (PGAP v.4.3) (Tatusova et al., 2016).

The OrthoANI package (Yoon et al., 2017) was used to measure the intra- and inter-species genome similarities by average nucleotide identity (ANI) among the assembled draft genomes. Further, FastANI (Jain et al., 2018) through DFAST (Tanizawa et al., 2018) was performed against 13000 prokaryotic reference genomes from NCBI to assess the taxonomic identity. It was also measured based on BLAST+ (ANIb) and MUMmer (ANIm) using JSpeciesWS (Richter et al., 2016).

The relatedness of our strains to *Legionella*-known type strains was further analyzed by applying a digital DNA–DNA hybridization (dDDH) to the closest related strain based on the previous ANI outcome. The method was implemented via the Genome-to-Genome Distance Calculator 2.1 (GGDC) web service (http://ggdc.dsmz.de), retaining default parameters, using BLAST+ (Camacho et al., 2009) as a local alignment tool. The GGDC uses a Genome Blast Distance Phylogeny (GBDP) to infer genome-to-genome distances between pairs of entirely or partially sequenced genomes. Below the similarity threshold of the *in silico* DDH (70%) and the ANI analysis (95%), two strains are considered distinct species (Meier-Kolthoff et al., 2013; Kim et al., 2014).

## Results

### *Legionella* spp. Isolation and Features

Starting from 2012, the *Legionella* environmental monitoring returned mostly negative results (<50 colony formant unit (cfu)/L). Only two samples were positive for *Legionella* in March 2015 and 2016 with a concentration of 200 and 250 cfu/L, respectively. The samples were collected from the shower and toilet showerhead in two different inpatient rooms, both located on the ground floor. The water temperature measured was 45.8 and 44.0°C, with a disinfectant residue of 20 mg/L. Regarding the colonies' morphology, they are small (about 3 mm), gray-white, with a round shape, and well compact. Concerning the identification, both isolates grew well on BCYE Cys+ and showed a quick positive reaction to the latex agglutination test for non-*Lp* species.

**Figure 2:**
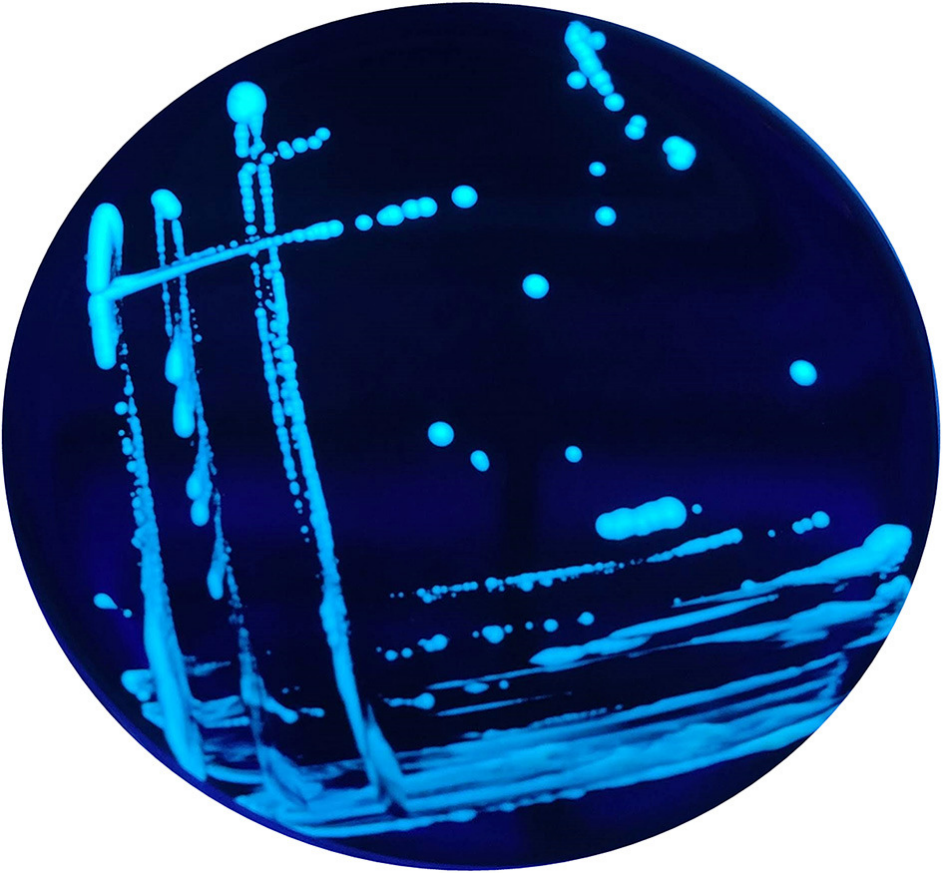
Blue-white autofluorescence of the non-*Lp* isolates grown on BCYE Cys+, found in 2015 and 2016, under Woods lamp (365 nm).

In addition, when exposed under a Woods lamp (long-wavelength UV light at 365 nm), they showed blue-white autofluorescence (Figure 2).

In particular, for further analysis, the strain isolated in 2015 was called 8cVS16, and the second one isolated in 2016 was named 9fVS26.

Considering that no other samples evaluated in the same year and during the subsequent monitoring yielded positive results for *Legionella*, the contamination found may be considered point-source contamination. Instead, the two strains were no longer detected in the water distribution system until December 2021, which was when the last sampling occurred.

### MALDI-TOF MS Results

The MALDI Biotyper system identified both strains with a low confidence score (yellow color) as *Legionella anisa*. In particular, the score returned for 8cVS16 was 1.76 and for 9fVS26 was 1.78.

**Figure 3:**
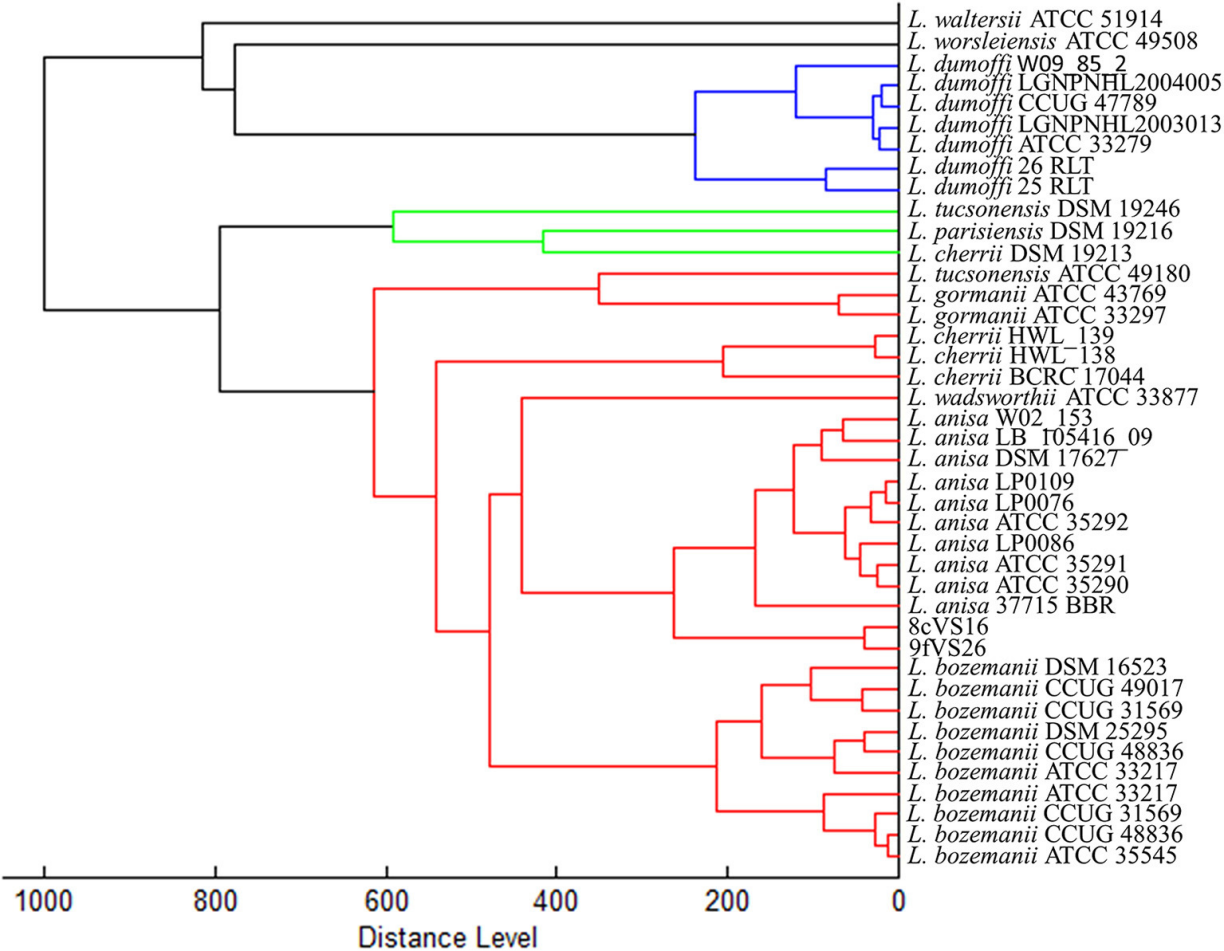
Dendrogram developed by HCA for 8cVS16 and 9fVS26 and related *Legionella* reference strains.

The dendrogram based on HCA, elaborated on the most closed *Legionella* species present in the instrument database, displays the 8cVS16 and 9fVS16 strains closed to each other and regrouped in one clade. This clade was well-separated by the two strictly related clades, represented by *L. anisa* and *Legionella bozemanii* reference strains (Figure 3). Therefore, the strains appeared very close to *L. anisa*, confirming the results returned by MALDI Biotyper software.

### *mip* and *rpoB* Results

BLAST research on NCBI and the ESGLI database returned the best match for both strains *L. anisa*, reference strain ATCC 35292 (GenBank accession number GCA_900639785.1), with similarities of 96.7 and 92.4% for *mip* and *rpoB*, respectively.

### Phylogenetic Analysis Results

**Figure 4:**
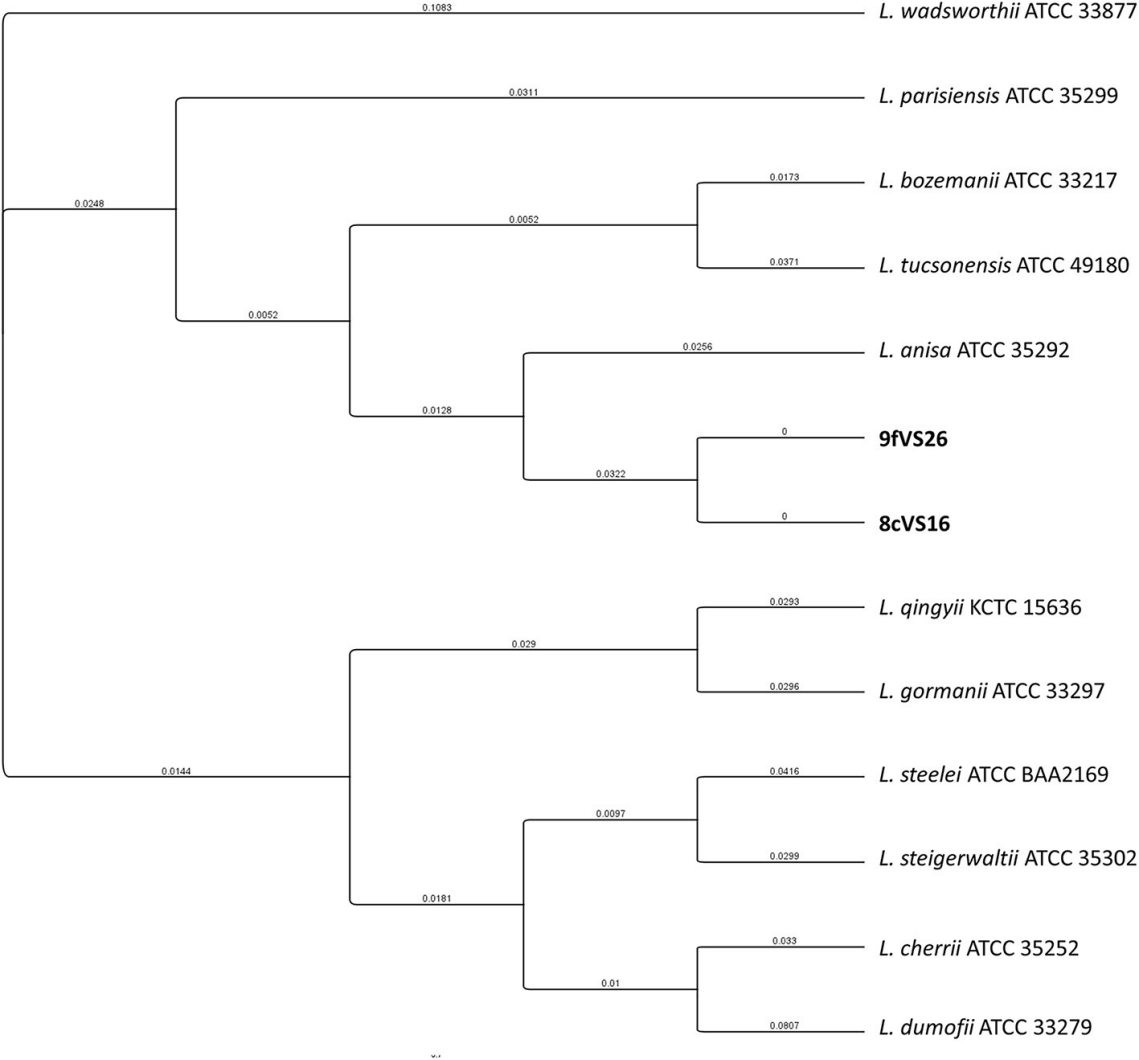
Phylogenetic tree of the two strains (8cVS16 and 9fVS26) and closely related *Legionella* species based on concatenation of two genes (*mip* and *rpoB*). Branch lengths are transformed to be equal like a cladogram. Branch labels display the substitutions per site.

The concatenated tree elaborated from *mip* and *rpo*B sequence genes (Figure 4) revealed the presence of two main clades, each containing four different subclades and each representing several *Legionella* species. Interestingly, the 8cVS16 and 9fVS26 strains were collocated into a monophyletic group comprising *L. anisa, Legionella tucsonensis*, and *L. bozemanii* strains. Moreover, it was particularly evident that they were strictly related to each other, forming a short branch representing a sister clade with the *L. anisa* ATCC 35292.

### WGS Analysis Results

All the results obtained by the WGS analysis are summarized in Table 1. Briefly, the overall lengths of the genomes for 8cVS16 and 9fVS26, respectively, were 3,906,083 and 3,906,1003 bp, with a GC content of 38.2% for both.

**Table 1:** Genome statistics data from NCBI and BUSCO quality analysis.

**Attribute**	**Data for strain**	
	8cVS16	9fVS26
No. of raw reads	1,787,078	1,952,986
Avg read length (bp)	256	259
Coverage (×)	115	127
Total Length (bp)	3,906,083	3,906,100
No. of contigs	7	10
GC Content (%)	38.2	38.2
N_50_ (bp)	855,940	858,038
No. of coding sequences	3,362	3,360
No. of rRNAs	6	6
No. of tRNAs	42	42
**BUSCO results [% (no. of genes)]**	**Data for strain:**	
	8cVS16	9fVS26
Complete	95.2 (118)	95.2 (118)
Single-copy complete	95.2 (118)	95.2 (118)
Duplicated complete	0.0 (0)	0.0 (0)
Fragmented	0.8 (1)	0.8 (1)
Missing	4.0 (5)	4.0 (5)
Total no. of BUSCO genes	124	124

In particular, BUSCO analysis, performed to evaluate the completeness of the two genome assemblies, indicates that the two genomes are near-complete, with a percentage of 95.2%.

Through the NCBI PGAP annotation required by GenBank to submit the draft genome, the following accession numbers were given: SRR17223244 and JAJTND000000000 for 8cVS16 and SRR17223245 and JAJSPM000000000 for 9fVS26.

Comparison, obtained from the OrthoANI package used to measure the intra- and inter-species genome similarities, returned the following values: 99.98% between 8cVS16 and 9fVS26, confirming that the two strains belong to the same species and are identical to each other.

The taxonomic identity, obtained by FastANI, returned an identity percentage of the closest strain for both 8cVS16 and 9fVS26. This strain was *L. anisa* strain WA-316-C3 (ATCC 35292) (GCA_900639785.1) with 90.74% ANI.

The analysis, performed by JSpeciesWS, confirmed the FastANI results with the following values: both 8cVS16 and 9fVS26 ANIb 90.08% compared with *L. anisa* WA-316-C3 (ATCC 35292), and ANIm 91.55% with *L. anisa* WA-316-C3 (ATCC 35292) for both 8cVS16 and 9fVS26 strains.

The analysis of our strains' relatedness to known types of strains using a digital DNA-DNA hybridization (dDDH) yielded the following results: a DDH value (generalized linear model (GLM)-based) of 43% for both 8cVS16 and 9fVS26 and a probability that DDH > 70% (i.e., same species) of 5.44% (via logistic regression).

Our results showed an ANI value of 90.74% and a dDDH value of 43%.

## Discussion

The discovery and identification of novel bacterial species are events of great relevance, especially when they occur during routine environmental surveillance programs. The identification of novel species is much more frequent in the clinical setting, thanks to the most advanced phenotypic and genotypic technologies. In contrast, laboratories involved in environmental surveillance routinely use only the standard culture method, so detection of a potentially novel species relies heavily on the researcher's and technician's experience. The results obtained in this study prove that it is possible to detect isolates, which are difficult to identify or could be misclassified with standard methods, by applying more sensitive techniques such as genotyping and WGS, even during routine environmental monitoring, carried out in self-surveillance.

Commonly, also in the widely studied and known WDS, the selective stress induced by changes in water characteristics (e.g., pressure, temperature, inorganic, and organic compounds), as well as the continuous disinfection treatments, may occur during the selection of a novel species, which is never identified before (Mcdonnell and Russell, 1999; Girolamini et al., 2021a, 2022).

The HCF monitored, from 2012, did not show *Legionella* contamination, except for 2 years (2015 and 2016). During our surveillance, only one sample per year was found positive, with non*-Lp* species concentration over the level of risk fixed to <100 cfu/L. A continuous disinfection treatment (hydrogen peroxide and silver salt) was installed at the time of facility opening, considering the size of the facility and the characteristics of the patients (e.g., elderly, with chronic and immunocompromised diseases). According to other studies, the continuous dosage of this disinfectant can control *Legionella* proliferation, although, from HCS opening, *Legionella* was never detected (Lin et al., 2011). Therefore, we cannot know whether *Legionella* was always absent due to the presence of disinfectant, or whether the WDS characteristics and the fact that the structure was newly built, contributed to creating a “*Legionella* free” environment. The presence of punctual contamination in two outlets, during 10 years of monitoring, both on flexible shower tubes, which are poorly used, due to the presence of non-self-sufficient patients, suggests that probably these pipelines constituted a niche with a biofilm, were able to protect, and would promote *Legionella* survival (Storey et al., 2004; Kaplan, 2010; Mahapatra et al., 2015). Moreover, in these types of outlets, as in the showerhead tubes, it is difficult to undertake cleaning and maintenance practices due to the small size of pipelines. The substitution of the showerhead flexible tubes, performed after the communication of *Legionella* presence, could explain the absence of *Legionella* detection to date.

It is not a matter to be neglected, as already demonstrated, that the continuous supply of chemical substances in WDS could promote disinfection resistance of bacterial species and the selection of novel ones (Mcdonnell and Russell, 1999; Girolamini et al., 2021a, 2022). We have already demonstrated in other facilities how the disinfectant based on peroxide hydrogen and silver salt in the same environment produces a different action on *Legionella*, promoting *L. anisa*'s resistance despite the high efficiency on *Lp*1 (Farhat et al., 2012; Girolamini et al., 2019, 2021b).

The culture technique, which is essential for the isolation, is not effective in correctly recognizing these novel isolates when combined with the common and rapid identification methods (e.g., agglutination test). Our results clearly showed that the two isolates presented features similar to well-known non-*Lp* species (e.g., *L. anisa, L. bozemanii*, and *Legionella gormanii*) and showed a positive agglutination reaction to multiple species latex reagents. Moreover, considering also the MALDI-TOF MS results, they could be misclassified as *L. anisa*.

In particular, the agglutination test has shown a positive result for non-*Lp* species antibodies and the phenotypic-proteomics analysis, provided by MALDI-TOF Biotyper, has identified the two strains as *L. anisa* with a low-confidential interval (yellow score), although the software can properly identify this species by assigning a green score, since the presence of its spectra in the instrument database. The results obtained were in accordance with the previous study (Moliner et al., 2010; Gaia et al., 2011; Svarrer and Uldum, 2012; Pascale et al., 2020), confirming the ability of MALDI-TOF MS to classify all the *Legionella* species contained in the database. The low confidence score returned for our isolates could be explained by the differences that 8cVS16 and 9fVS26 spectra showed with the *L. anisa* reference spectra contained in the instrument database. So, we can suppose that the differences at the genomic level were also reflected at the ribosomal protein level, which is the target of MALDI-TOF MS technique. These differences found at the proteomics level appear more evident in the dendrogram returned by HCA, where it is possible to observe how 8cVS16 and 9fVS26 form a separate clade with respect to the two main clades represented by *L. anisa* and *L. bozemanii*.

Starting from these considerations, the sequencing of *Legionella* characteristics genes is essential to obtain discrimination at interspecies and intraspecies levels.

Regarding the genomic analysis, *mip* and *rpoB* gene sequencing confirmed the previous results. The two isolates were associated with *L. anisa*, showing an identity percentage of 96.7 and 92.4%, for *mip* and *rpoB*, respectively, below the established threshold (Ratcliff et al., 1998; Ko et al., 2002; Adékambi et al., 2009; Pascale et al., 2021). The phylogenetic tree well-points out these differences, showing how the two isolates, despite their proximity to the *L. anisa* clade, represent a single subclade that needs to be further investigated.

The discrepancies found in sequences identity percentage have led us to investigate in more depth by applying the WGS technique. The genomes of 8cVS16 and 9fVS26 were then compared with the closest genome returned by FASTANI belonging to *L. anisa* (ATCC 35292). Although the whole-genome sequences for both isolates were almost completed, with a coverage of 95.2%, the ANI analysis returned a result of 90.74% and the dDDH analysis reported a value of 43% compared to the *L. anisa* ATCC 35292. Considering that the percentage obtained was under the established similarity thresholds (95 and 70%, respectively) (Meier-Kolthoff et al., 2013; Kim et al., 2014), we can consider the two isolates found belonging to a novel *Legionella* species.

More detailed studies on (i) morphology, including flagellar structures, (ii) *Legionella* growth tests at various temperatures and on culture media, (iii) biochemical, and (iv) antibiotic susceptibility tests will be the next steps in obtaining isolates characterization, resulting in the deposit of the type strain in culture collections and the most complete description of the novel species.

This study can be considered as a support tool for all laboratories that, during the phases of environmental surveillance, may find isolates with similar characteristics to the best-known species but with discrepancies in results. These laboratories are encouraged not to stop at the common identification tests but to continue the investigations in order to discover novel isolates and understand the dynamics that led to their development. The results of this study should not surprise us should we consider that the environment is undergoing profound changes (e.g., global warming), which can lead to an increase in temperature in both natural and artificial reservoirs. Moreover, the increasingly widespread use of disinfectants as a preventive strategy, other than changes in water characteristics and pipeline materials, leads to a strong impact on the selection of bacteria and the resistance development.

Therefore, acquiring extensive knowledge of these reservoirs and the events that can promote changes in the ecological niche of bacteria can help to prevent their spread in the man-made environment and contain the occurrence of cases, clusters, or outbreaks. In this context, environmental surveillance is critical, as is a proper risk assessment plan that takes into account changes in Legionella contamination over time. The latter will primarily focus on correct isolate identification and characterisation, as well as novel approaches able to recognise non-Lp species with unknown rates of infection and pathogenicity other than antibiotic resistance.

## Data Availability Statement

The datasets presented in this study can be found in online repositories. The names of the repository/repositories and accession number(s) can be found in the article/supplementary material.

## Author Contributions

LG, MRP, SSa, and SC conceived and designed the experiments and wrote the paper. LG, MRP, MM, FM, and SSp performed sample collection and culture experiments. MO performed the whole-genome sequencing. AG performed gene sequencing. LG, SSa, and MO performed the bioinformatics analysis. The manuscript has been read and approved by all named authors. All authors contributed to the article and approved the submitted version.

## Conflict of Interest

The authors declare that the research was conducted in the absence of any commercial or financial relationships that could be construed as a potential conflict of interest.

## Publisher's Note

All claims expressed in this article are solely those of the authors and do not necessarily represent those of their affiliated organizations, or those of the publisher, the editors and the reviewers. Any product that may be evaluated in this article, or claim that may be made by its manufacturer, is not guaranteed or endorsed by the publisher.
